# First description of an acinic cell carcinoma of the breast in a *BRCA1* mutation carrier: a case report

**DOI:** 10.1186/1471-2407-13-46

**Published:** 2013-02-01

**Authors:** Carla B Ripamonti, Mara Colombo, Patrizia Mondini, Manoukian Siranoush, Bernard Peissel, Loris Bernard, Paolo Radice, Maria Luisa Carcangiu

**Affiliations:** 1 Unit of Molecular Bases of Genetic Risk and Genetic Testing, Department of Preventive and Predictive Medicine, Fondazione IRCCS Istituto Nazionale dei Tumori, Milan, Italy; 2 Unit of Medical Genetics, Department of Preventive and Predictive Medicine, Fondazione IRCCS Istituto Nazionale dei Tumori, Milan, Italy; 3 Division of Experimental Oncology, Istituto Europeo di Oncologia, Milan, Italy; 4 Cogentech, Cancer Genetic Test Laboratory, IFOM-IEO Campus, Milan, Italy; 5 IFOM, Fondazione Istituto FIRC di Oncologia Molecolare, Milan, Italy; 6 Anatomic Pathology Unit 1, Department of Pathology and Laboratory Medicine, Fondazione IRCCS Istituto Nazionale dei Tumori, Milan, Italy

**Keywords:** Acinic cell carcinoma, Breast cancer, *BRCA1*, Triple negative, *TP53* mutation

## Abstract

**Background:**

Acinic cell carcinoma (ACC) is a rare malignant epithelial neoplasm characterized by the presence of malignant tubular acinar exocrine gland structures. Diagnosis is generally made in salivary glands and in the pancreas. ACC of the breast has been reported in few cases only. Carriers of inherited mutations in the *BRCA1* gene are prone to the development of breast cancer, mainly invasive ductal or medullary type carcinomas. We describe for the first time a *BRCA1* mutation carrier with a diagnosis of ACC of the breast.

**Case presentation:**

The patient developed an invasive ductal carcinoma (IDC) at the age of 40 years and an ACC in the contralateral breast at 44 years. Immunohistochemical examination of the ACC revealed a triple negative status (i.e., negativity for estrogen receptor, progesterone receptor and HER2 protein) and positivity for p53. Using a combination of loss of heterozygosity (LOH) and sequencing analyses, the loss of the wild-type *BRCA1* allele was detected in both the ACC and the IDC. In addition, two different somatic *TP53* mutations, one in the ACC only and another one in the IDC only, were observed.

**Conclusion:**

Both the immunohistochemical and molecular features observed in the ACC are typical of *BRCA1*-associated breast cancers and suggest an involvement of the patient’s germline mutation in the disease. The occurrence of rare histological types of breast cancers, including malignant phyllodes tumor, atypical medullary carcinoma and metaplastic carcinoma, in *BRCA1* mutation carriers has been already reported. Our findings further broaden the spectrum of *BRCA1*-associated breast malignancies.

## Background

Acinic cell carcinoma (ACC) is a rare histological type of malignant epithelial neoplasm exhibiting acinic cell differentiation. The typical locations are major and minor salivary glands and pancreas, where it accounts for approximately 10% and 1% of all cancers, respectively [1-3]. ACC has been occasionally observed in other organs, including lung, stomach, liver, retroperitoneum, lacrimal glands and breast [4-19]. ACC of the breast was first described by Roncaroli *et al*. in a 42-year-old woman who complained of a palpable lump that had rapidly increased in size and appeared at mammography as a well defined lesion containing scattered granular calcifications [10]. Since then, a few additional cases have been reported in patients aged between 23 and 80 years, including a male subject [11-19]. Morphologically, ACC of the breast resembles the homonymous salivary gland tumor due to the formation of tubular acinar gland structures delimited by a thin layer of connective tissue. Two distinct patterns of growth have been described. The first is solid or nesting, poorly circumscribed and infiltrating, often accompanied by focal necrosis. The second pattern is characterized by the formation of acinar, tubular, microglandular and microcystic structures. The neoplastic cells are polygonal or round, with an amphophilic cytoplasm containing coarse brightly eosinophilic granules. The glandular lumina contain an amorphous eosinophilic material [20,21].

Approximately 10% of breast cancers show a hereditary predisposition. It is estimated that nearly 20% to 30% of these hereditary tumors are linked to germline mutations in either the *BRCA1* or *BRCA2* genes [22,23]. *BRCA1* mutation carriers face a lifetime risk to develop breast cancer that ranges from 57% to 65% [24,25]. The pathological features of breast tumors arising in *BRCA1* mutation carriers have been extensively described. They are mostly high grade invasive ductal not otherwise specified (NOS) or medullary type carcinomas. High mitotic index, pushing margins, lymphocytic infiltration and areas of necrosis are frequent findings. Immunoreactivity for estrogen receptor (ER), progesterone receptor (PgR) and for the human epidermal growth factor receptor 2 (HER2) is usually absent [26-28]. This profile, defined as “triple negative” (TN), has been shown to be highly predictive of the *BRCA1* mutation carrier status [29].

*BRCA1* is a tumor suppressor gene involved in the control of cell cycle progression and of DNA double strand break (DSB) repair. The vast majority of breast tumors developing in *BRCA1* carriers shows inactivation of the wild-type allele by either somatic genetic (mutations or loss of heterozygosity (LOH)) or epigenetic (promoter hypermethylation) changes [30]. These occurrences lead to the absence of functional BRCA1 protein and, thus, to genetic instability and tumor development. In addition, breast tumors in *BRCA1* mutation carriers frequently show mutations of the *TP53* gene and/or abnormal expression of the corresponding protein p53 [31,32].

In this study, we provide the first pathological, immunohistochemical and molecular characterization of a breast tumor of the ACC type developed in a *BRCA1* mutation carrier.

## Case presentation

### Clinical course and pathological features

**Figure 1:**
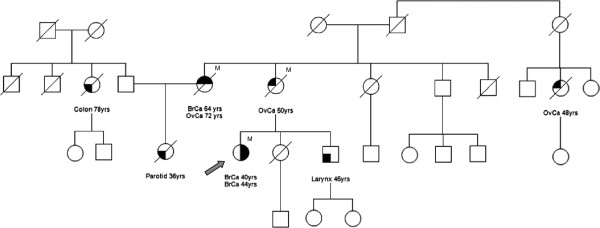
**Family tree of the patient (indicated by the arrow).** Individuals affected with cancer, ages at diagnosis and *BRCA1* mutation carriers (M) are indicated. No other family member could be tested for the presence of the mutation. BrCa, breast cancer; OvCa, ovarian cancer.

The patient is a 45-year-old woman with a familial history of breast and ovarian carcinomas and other cancers (Figure 1). Her mother died at the age of 50 years from ovarian cancer, and her maternal aunt developed breast cancer at 64 years and ovarian cancer at 72 years. The daughter of the latter patient died at 44 years from a metastatic malignant tumor, consistent with a salivary gland origin. Genetic testing of the patient’s maternal aunt identified a constitutional *BRCA1* mutation, c.4484 G>T, causing the skipping of exon 14 [33,34]. Subsequently, the presence of this mutation was ascertained in the subject of this report at the age of 36 years, when still in good health.

Following this finding, clinical examination and instrumental surveillance with mammography, magnetic resonance imaging (MRI) and ultrasonography were initiated according to the institutional protocols for BRCA gene mutation carriers. At the age of 40 years, a poorly differentiated invasive ductal carcinoma (IDC), measuring 25 mm in diameter, was diagnosed in the upper outer quadrant of the right breast. The pathology report described positive immunostaining for ER and PgR and negativity for HER2. A radical mastectomy with sentinel lymph node biopsy was performed. According to the pathological TNM (tumor, node, metastasis) staging system, the tumor was pT2, pN0. The patient received adjuvant chemotherapy with adriamycin, cyclophosphamide, methotrexate and 5-fluorouracil, and hormonal therapy with tamoxifen and luteinizing-hormone-releasing hormone (LH-RH) analogue. At the age of 41 years, two left axillary lymph nodes were biopsied and reported as reactive and negative for metastatic tumor. One year later, the patient underwent prophylactic salpingo-oophorectomy and the LH-RH analogue was stopped, whereas the tamoxifen therapy was switched to aromatase inhibitors.

**Figure 2:**
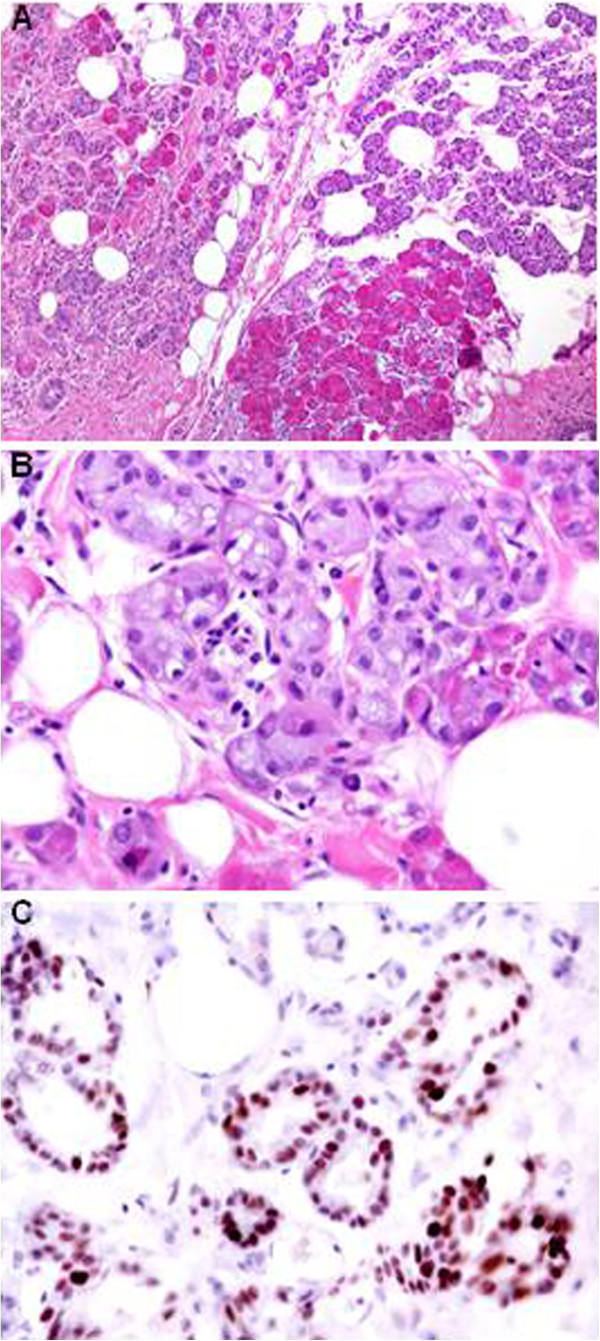
**Haematoxylin and Eosin staining (A, B) and *p53 *immunostains (C) of ACC [x10 (A) and x40 (B, C) magnifications].**

At the age of 44 years, a breast MRI revealed a solid mass with pushing margins in the upper outer quadrant of the left breast. A mammary resection (lumpectomy) was carried out, and the histological diagnosis was made of ACC with a microglandular pattern of growth. On gross examination, the tumor appeared as an ill-defined yellowish firm area measuring 13 mm in its largest dimension. Microscopically, it was characterized by a diffuse tightly packed proliferation of small acinar and glandular structures, frequently intermixed with solid nests of markedly eosinophilic larger cells. The acinar and glandular structures were made of round cells with a finely granular, weakly basophilic, or clear cytoplasm resembling those of acinar cells of salivary glands. The eosinophilic cells showed bright eosinophilic coarse granules resembling those of intestinal Paneth cells (Figure 2A,B).

Immunohistochemical stainings were performed using the streptavidin-biotin peroxidase method, in accordance with the manufacturer's instructions (Table 1). The neoplastic cells were immunohistochemically positive for the S100 protein, epithelial membrane antigen (EMA), p53 (Figure 2C) and alpha*-*1*-*antichymotrypsin (AACT) whereas maspin, gross cystic disease fluid protein-15 (GCDFP), ER, PgR and HER2 were negative. Immunostains for actin and calponin did not show myoepithelial differentiation.

**Table 1:** Primary antibodies and dilutions used for the immunohistochemical analyses and results

**Antibody to**	**Manufacturer**	**Dilution**	**Results**
ER	Ventana	ready-to-use	Negative
PgR	Ventana	ready-to-use	Negative
HER2	Dako	1:1000	Negative
S100 protein	Dako	1:2000	Positive
EMA	Ventana	ready-to-use	Positive
GCDFP15	Signet	1:20	Negative
P53	Novocastra	1:200	Positive
AACT	Dako	1:1000	Positive
Actin	Dako	1:400	Negative
Maspin	Pharmingen	1:500	Negative
Calponin	BioGenex	1:400	Negative

ER, estrogen receptor; PgR, progesterone receptor; HER2, human epidermal growth factor receptor 2; EMA, epithelial membrane antigen; GCDFP15, gross cystic disease fluid protein-15; AACT, alpha*-*1*-*antichymotrypsin.

Two months later, a radical mastectomy was performed. No residual tumor was found in the remaining parenchymal tissue or in the two microscopically examined lymph nodes. The patient is alive and well 19 months after the surgery, with no evidence of disease and still undergoing hormonal therapy.

A written consent approved by the Ethical Committee of the Fondazione IRCCS Istituto Nazionale dei Tumori, was obtained from the patient to the use of her biological samples for research purposes.

### Genetic investigations

**Figure 3:**
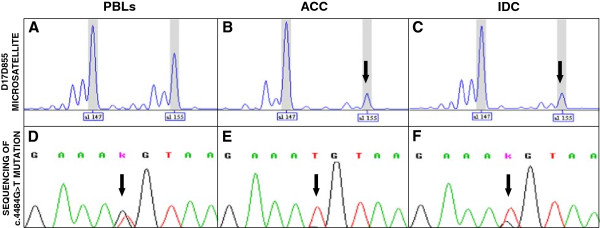
**Assessment of the loss of the wild-type *BRCA1 *allele in tumor DNA. A-C)** The two *D17S855* alleles (al_147 and al_155) present in the peripheral blood leukocytes (PBLs) DNA of the patient are indicated. A strong reduction of the peak corresponding to the al_155 allele is observed in both the ACC and the IDC DNA (indicated by the arrows). **D-F)***BRCA1* sequence analysis. The site of the germline c.4484 G>T mutation is indicated by the arrows. A reduction of the wild-type G allele is visible in both the ACC and IDC DNA.

Formaldehyde-fixed, paraffin-embedded surgical specimens of both the ACC and the IDC were retrieved and reviewed by the pathologist. Tumor areas were manually microdissected. DNA was extracted from tumor and normal (peripheral blood leukocytes; PBLs) tissues using commercial kits (QIAGEN). The region corresponding to the *BRCA1*-associated *D17S855* polymorphic microsatellite marker was amplified by polymerase chain reaction (PCR) from the ACC and normal DNA, using a 5^′^-end fluorescent dye labeled forward primer (VWR International). The amplification products were separated by capillary electrophoresis on an ABI 3130 Genetic Analyzer (Applied Biosystems) and analyzed using the Gene Mapper 4.0 software (Applied Biosystems). The ratio between the peak area of the larger allele and that of the smaller allele in tumor DNA (Figure 3B) was calculated after normalization on the corresponding ratio in the normal DNA (Figure 3A). LOH was assessed in the tumor sample where a strong reduction (ca. 70%) of the peak ratio was observed as compared to normal DNA. To evaluate which *BRCA1* allele was lost, we performed the sequence analysis of the genomic region encompassing the *BRCA1* constitutional mutation in normal and ACC DNA. The sequence reactions were performed with the ABI PRISM® Big Dye Terminator Cycle Sequencing Kit (Applied Biosystems), run on an ABI 3130 Genetic Analyzer and examined using the Sequencing Analysis Software (Applied Biosystems). A strong reduction of the constitutionally wild-type allele (G) was observed in the ACC (Figure 3E), compared to normal DNA (Figure 3D). Therefore, the ACC presented a bi-allelic inactivation of the *BRCA1* gene due to the constitutional mutation coupled with the somatic loss of the wild-type allele.

**Figure 4:**
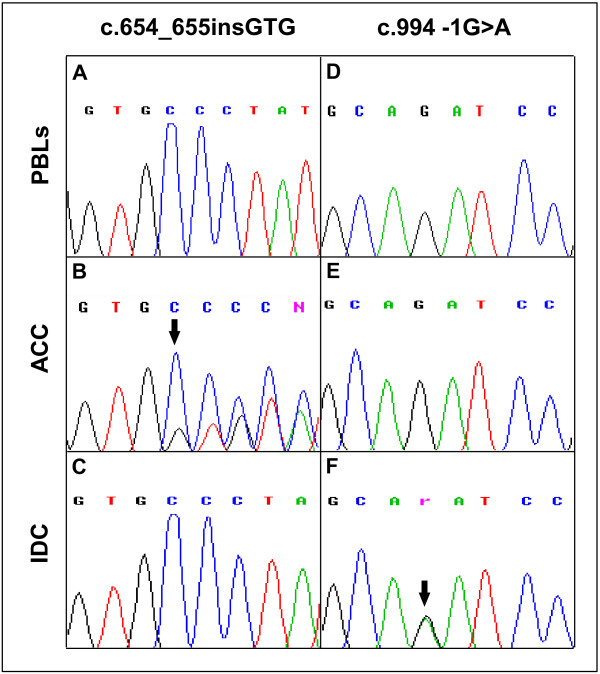
**TP53 sequence analysis. A-C**) DNA sequences showing the presence of the c.654_655insGTG mutation in the ACC and its absence in the IDC and PBL DNA. **D-F**) DNA sequences showing the presence of the c.994 -1 G>A mutation in the ICD and its absence in the ACC and PBL DNA. The position of the mutations is indicated by the arrows. PBLs, peripheral blood leukocytes.

The sequencing of coding exons (2 to 11) of the *TP53* gene in the ACC identified the c.654_655insGTG mutation (Figure 4B), which was not present in the normal DNA (Figure 4A). This mutation was previously described in a case of Burkitt lymphoma [35].

Using the above described approaches, loss of the wild-type *BRCA1* allele (Figures 3C, 3F) and a *TP53* mutation (c.994-1 G>A) (Figure 4F) were also observed in the IDC sample. The *TP53* mutation was not present in both the normal (Figure 4D) and the ACC (Figure 4E) DNA. Conversely, the *TP53* mutation observed in the ACC was absent in the IDC (Figure 4C).

## Discussion

A few studies have recently documented the occurrence of ACCs in patients with hereditary susceptibility to cancer. Five reports have been published describing the development of ACCs of the pancreas in subjects carrying germline mutations in genes predisposing to breast and colon cancers. More specifically, a pancreatic ACC was reported in a *BRCA1* male mutation carrier with a family history positive for early-onset breast cancers. The proband had a previous history of papillary renal and colon cancers and was affected with acromegaly [36]. Skoulidis *et al*. described ACCs of the pancreas in three subjects with *BRCA2* germline mutations and detected LOH at the *BRCA2* locus in the tumors [37]. Furthermore, the diagnosis of pancreatic ACC was made in a 46-year-old male affected with liposarcoma, polyps and hamartomas of the colon, who carried a germline mutation in *STK11*/*LKB,* the gene causing the Peutz-Jeghers syndrome. By LOH analysis, the authors assessed a link between the loss of the wild-type *STK11/LKB1* allele and the onset of the ACC [38]. Interestingly, no *STK11/LKB1* mutation or altered RNA expression were, by contrast, found in five sporadic ACCs of the pancreas [39]. A metastatic ACC of the pancreas was described in a female patient carrying a mutation in *MSH6*, one of the genes responsible for the Lynch syndrome. The patient had received previous diagnoses of invasive breast cancer, and sebaceous and basal cell carcinomas of the skin [40]. Finally, germline mutations of *PRKAR1A*, the gene responsible for the Carney complex, were observed in two patients with pancreatic ACCs. LOH at the gene locus and absence of the corresponding protein expression were observed in tumor cells [41].

An ACC of the retromolar trigone region was described in a 35-year-old patient with a clinical diagnosis of Cowden disease and a genetic analysis positive for a pathogenic mutation in *PTEN*[42]. Cowden disease is characterized by the presence of multiple hamartomas, a type of malformation mainly affecting the gastrointestinal tract, and an increased susceptibility to breast, thyroid and endometrial cancers [43].

To the best of our knowledge, our report is the first describing a primary ACC of the mammary gland in a *BRCA1* mutation carrier. Histologically, the tumor displayed two populations, one characterized by a microglandular pattern of growth and the other consisting of solid nests of cells. The glandular cells were characterized by basophilic cytoplasm, while the latter showed eosinophilic cytoplasmic granules. The results of immunohistochemical analysis were in accordance with previous reports and confirmed the diagnosis of ACC [11-19]. In addition, the tumor exhibited a TN phenotype, in agreement with four out of the five previous reports of ACCs of the breast for which the results of ER, PgR and HER2 stains were available [13,15,17-19].

Molecular characterization detected the loss of the *BRCA1* wild-type allele and a *TP53* somatic mutation in tumor DNA. The involvement of *TP53* in tumor development was confirmed by immunohistochemistry that showed positivity for p53*.* By contrast, the only previously reported ACC of the breast that had been examined for p53 expression tested negative on immunostaining [13]. We also investigated for LOH at *BRCA1* locus and for *TP53* mutations the DNA extracted from the IDC that the patient had developed few years before the diagnosis of ACC. Like the ACC, the IDC displayed loss of the *BRCA1* wild type allele and the somatic *TP53* mutation. However, this mutation was different from the one detected in the ACC, indicating a diverse origin of the two tumors. Our results are consistent with the observation that somatic alterations of *BRCA1* and *TP53* are frequent in breast cancers, particularly those of the TN phenotype [44,45]. Both genes are key players in the control of DNA damage response and their impairment leads to tumor development by inducing, among other mechanisms, genomic instability.

Our findings are consistent with previous studies reporting LOH at *BRCA1* locus and *TP53* abnormalities as frequent events in breast cancers occurring in *BRCA1* mutation carriers [31,32]. Therefore, we may argue a causal role of the genetic background of the patient, which made her prone to breast cancer, not only in the development of the IDC, but also in the rare phenotypic expression of the ACC. Interestingly, a considerable proportion (7/14 = 50%) of the previously documented cases of ACC of the breast were of early-onset (age at diagnosis <50 years) [10-12,14-16,18]. This observation suggests the involvement of hereditary factors in at least a fraction of these patients.

Atypical and unusual histologic features of breast cancers in *BRCA1* mutation carriers have been reported. A malignant phyllodes tumor was observed in a 43-years-old patient, who tested positive for the c.5095C>T (p.R1699W) missense pathogenic mutation [34,46]. In addition, Ashida *et al.* reported an atypical medullary carcinoma of the breast with cartilaginous metaplasia in a young patient with a nonsense mutation in exon 5 [47]. The association of *BRCA1* mutations with other histotypes, commonly grouped under the term of metaplastic breast carcinomas (MBC) and including squamous, adenosquamous and tumors with biphasic morphology carcinomas (so-called carcinosarcomas), was also described. Breuer *et al.* reported on a young woman, carrying the c.181 T>G (p.C61G) mutation, who had developed bilateral asynchronous squamous breast cancers at 25 and at 28 years of age [48]. Suspitsin *et al*. described a mixed epithelial/mesenchimal metaplastic carcinoma that was diagnosed in a 35-year-old female carrier of the c.5266dupC mutation. The molecular analysis revealed the loss of the wild-type *BRCA1* allele in both the epithelial and the mesenchymal components of the tumor, supporting the role of *BRCA1* in its development [49]. A low-grade adenosquamous carcinoma of the breast described by Noel *et al.* was diagnosed in a 49-year-old woman with a previous diagnosis of IDC and the presence of the c.66dupA mutation [50]. Finally, Rashid *et al.* reported a biphasic carcinoma composed of sarcomatous and malignant epithelial components in a 22-year-old proband who carried the c.68_69delAG mutation [51]. With the single exception of the study of Suspitsin *et al*., the above mentioned reports described a “basal like” phenotype of the tumor tissues, on the basis of the TN status and the positivity by immunohystochemistry of the basal cytokeratin CK5/6. In fact, the majority of *BRCA1*-related breast cancers express basal cytokeratins [27] and the discovery of this status in the MBCs reported to date is in favor of the involvement of *BRCA1* in the tumor development.

## Conclusions

We described the first case of a *BRCA1* mutation carrier affected with an ACC of the breast, a very rare histological subtype of mammary tumor. Immunohistochemical analyses and molecular investigations of the ACC provided evidences suggestive of an involvement of the constitutional *BRCA1* mutation in the pathogenesis of disease. In addition to the present case, studies reporting on malignant phyllodes tumor, atypical medullary carcinoma and MBCs of the breast support the notion that rare histological types of breast cancers can occur in patients with mutations in *BRCA1* and that their development is mediated by the presence of such mutations. Further studies are needed to understand which additional factors contribute to make *BRCA1* mutation carriers prone to the expression of different breast tumor phenotypes.

### Consent

Written informed consent was obtained from the patient for publication of this Case report and any accompanying images. A copy of the written consent is available for review by the Editor of this journal.

## Competing interests

The authors declare that they have no competing interests.

## Authors’ contributions

RCB designed the study, carried out the molecular analyses and drafted the manuscript. CM helped to perform the sequencing analyses and to draw the figures. MP provided technical support. MS and PB retrieved and provided the clinical data. BL performed the *BRCA1* mutation screening. RP provided the final editing and approval of the manuscript for publishing. CML made the histological diagnosis, performed the immunohistochemical analyses and contributed to the writing of the manuscript. All authors read and approved the final manuscript.

## Pre-publication history

The pre-publication history for this paper can be accessed here:

http://www.biomedcentral.com/1471-2407/13/46/prepub
